# Stimulation of Chondrocyte and Bone Marrow Mesenchymal Stem Cell Chondrogenic Response by Polypyrrole and Polypyrrole/Gold Nanoparticles

**DOI:** 10.3390/polym15112571

**Published:** 2023-06-03

**Authors:** Ilona Uzieliene, Anton Popov, Viktorija Lisyte, Gabija Kugaudaite, Paulina Bialaglovyte, Raminta Vaiciuleviciute, Giedrius Kvederas, Eiva Bernotiene, Almira Ramanaviciene

**Affiliations:** 1 Department of Regenerative Medicine, State Research Institute Centre for Innovative Medicine, LT-08406 Vilnius, Lithuania; ilona.uzieliene@imcentras.lt (I.U.); gabija.kugaudaite@gmail.com (G.K.); paulinabalagovyte@gmail.com (P.B.); raminta.vaiciuleviciute@imcentras.lt (R.V.); eiva.bernotiene@imcentras.lt (E.B.); 2 Department of Immunology, State Research Institute Centre for Innovative Medicine, LT-08406 Vilnius, Lithuania; anton.popov@imcentras.lt; 3 NanoTechnas–Center on Nanotechnology and Materials Sciences, Faculty of Chemistry and Geosciences, Vilnius University, LT-03225 Vilnius, Lithuania; viktorija.lisyte@chgf.stud.vu.lt; 4 The Clinic of Rheumatology, Traumatology Orthopaedics and Reconstructive Surgery, Institute of Clinical Medicine of the Faculty of Medicine, Vilnius University, LT-03101 Vilnius, Lithuania; giedrius.kvederas@santa.lt; 5 VilniusTech Faculty of Fundamental Sciences, Department of Chemistry and Bioengineering, Vilnius Gediminas Technical University, LT-10221 Vilnius, Lithuania

**Keywords:** bone marrow mesenchymal stem cells, chondrocytes, chondrogenic differentiation, polypyrrole, polypyrrole/gold nanoparticles

## Abstract

Bone marrow mesenchymal stem cells (BMMSCs) possess a strong ability to differentiate into the chondrogenic lineage, which is important for cartilage regeneration. External stimuli, such as electrical stimulation (ES), are frequently studied for chondrogenic differentiation of BMMSCs; however, the application of conductive polymers such as polypyrrole (Ppy), has never been used for stimulating BMMSCs chondrogenesis in vitro before. Thus, the aim of this study was to evaluate the chondrogenic potential of human BMMSCs after stimulation with Ppy nanoparticles (Ppy NPs) and compare them to cartilage-derived chondrocytes. In this study, we tested Ppy NPs without and with 13 nm gold NPs (Ppy/Au) for BMMSCs and chondrocyte proliferation, viability, and chondrogenic differentiation for 21 days, without the use of ES. The results demonstrated significantly higher amounts of cartilage oligomeric matrix protein (COMP) in BMMSCs stimulated with Ppy and Ppy/Au NPs, as compared to the control. The expression of chondrogenic genes (*SOX9*, *ACAN*, *COL2A1*) in BMMSCs and chondrocytes were upregulated by Ppy and Ppy/Au NPs, as compared to controls. Histological staining with safranin-O indicated higher extracellular matrix production in Ppy and Ppy/Au NPs stimulated samples, as compared to controls. In conclusion, Ppy and Ppy/Au NPs stimulate BMMSC chondrogenic differentiation; however, BMMSCs were more responsive to Ppy, while chondrocytes possessed a stronger chondrogenic response to Ppy/Au NPs.

## 1. Introduction

Polypyrrole (Ppy) is a biocompatible polymer used in different biomedical applications, usually under electrical stimulation (ES). Ppy is mostly applied by covering specific layers/scaffolds or mixing it with hydrogels/electrospun fibers to stimulate cellular functions in vitro. This polymer can be obtained by electrochemical and chemical synthesis in the form of layers or micro- and nanoparticles (NPs) at neutral pH of aqueous solutions [1,2]. It has excellent biocompatibility, flexibility, and stability. Ppy is a potential tool in the delivery of drugs or target proteins and can be used either in combination with other compounds, such as organic molecules, or used alone as NPs [3,4]. Ppy structures are known to improve the rate of charge transfer across the cell membrane and stimulate the proliferation and differentiation of stem cells [5,6,7].

Nowadays, there is a huge interest in the use of Ppy and its derivatives for various tissue regeneration purposes, mostly to stimulate excitable cell differentiation towards muscle, cardiac and neural tissues under ES [2,8]. Additionally, many studies analyzed non-excitable cells, such as mesenchymal stem cells’ (MSCs) potential to differentiate into various tissue-like cells under ES using Ppy structures [6,9,10].

Bone marrow MSCs (BMMSCs) are considered one of the means for different tissue regenerative purposes, including poorly self-repairing tissues such as cartilage [11,12]. Articular cartilage is an avascular tissue, which contains primary cells, chondrocytes, responsible for tissue integrity. Once damaged, cartilage is challenging to repair and regenerate itself, often leading to the development of osteoarthritis (OA) [13]. BMMSCs possess a strong ability to differentiate into chondrocyte-like cells and their differentiation in vitro is stimulated using the specific chondrogenic medium, which includes transforming growth factor β3 (TGF-β3), important for chondrogenic gene expression [14]. ES is a frequent method and was previously applied for BMMSCs chondrogenic differentiation studies [15,16]. ES activates cells, depolarizes the plasma membrane, and improves the flow of calcium (Ca^2+^) ions important for chondrogenic differentiation, which stimulates the production of further cartilage tissue proteins [17,18]. However, to the best of our knowledge, chondrogenic differentiation of BMMSCs using Ppy NPs has never been studied before.

The aim of this study was to evaluate the chondrogenic potential of human BMMSCs after stimulation with Ppy NPs and compare them to cartilage-derived chondrocytes without the use of ES. In this study, we tested two types of NPs-Ppy NPs and Ppy combined with gold NPs (Ppy/Au) for BMMSCs and chondrocyte functions, including proliferation, viability, and chondrogenic capacity. Ppy/Au NPs were chosen due to their positive effects in drug or gene delivery, photothermal therapy, and other tissue regeneration-related studies made before [19,20]. The study focused on the investigation of BMMSCs chondrogenic differentiation capacity after incubating with Ppy and Ppy/Au NPs without the use of ES. According to the literature, Ppy itself supports some cellular functions even without additional application of ES, such as proliferation and differentiation [21,22]; therefore, we were interested to test the effects of its NPs on BMMSCs and chondrocyte functions, which will be important background data for the further experiments introducing ES for chondrogenic stimulation. Chondrocytes were used as control cells for a qualitative chondrogenic response. We synthesized Ppy and Ppy/Au NPs and applied them in BMMSCs and chondrocyte proliferation/viability testing, as well as in two chondrogenic differentiation models (2D and 3D).

## 2. Materials and Methods

### 2.1. Synthesis of Ppy and Ppy/Au NPs

The colloidal gold NPs (AuNPs) were obtained from hydrogen tetrachloroaurate trihydrate (HAuCl_4_ · 3H_2_O) according to the previous study [23]; 40 mL of 0.0125% (*w*/*v*) aqueous solution of HAuCl_4_ and 10 mL of a second aqueous solution consisting of 0.2% (*w*/*v*) sodium citrate and 0.00125% (*w*/*v*) tannic acid were heated separately to 60 °C. The solutions were mixed, stirred, heated to 95 °C, and incubated at this temperature for 5 min. AuNPs colloid was cooled at room temperature and stored in the dark at +4 °C. AuNPs were characterized using UV-VIS spectrophotometer Lambda 25 (PerkinElmer, Waltham, MA, USA) and high-resolution field emission scanning electron microscope SU-70 (Hitachi, Tokyo, Japan).

Ppy and Ppy/Au NPs were synthesized using an adapted previously published procedure [24]; 0.05 M citric buffer, pH 2, was prepared using citric acid monohydrate (Sigma Aldrich, Steinheim, Germany) and trisodium citrate dihydrate (Scharlau, Sentiment, Spain). Pyrrole (Alfa Aesar, Kandel, Germany) was distilled before use. Then, a 0.25 M pyrrole solution in citric buffer, pH 2, was prepared by stirring in an ultrasonic bath. Ppy NPs polymerization solution was obtained by adding H_2_O_2_ (Carl Roth, Karlsruhe, Germany) to a final concentration of 9 mM. In the case of Ppy/Au NPs, the preparation procedure was supplemented by adding 5 mL AuNPs colloid and mixing before H_2_O_2_ was introduced. Both polymerization solutions were left at 40 °C for 48 h. Ppy and Ppy/Au NPs were washed three times by centrifugation with distilled water. Obtained Ppy and Ppy/Au NPs were characterized using scanning electron microscopy (SEM).

### 2.2. BMMSCs and Chondrocyte Isolation and Culture

All procedures with the donor tissues were performed in accordance with the Bioethical Permission (No. 158200-14-741-257) and its supplemented version (Permission No. 158200-741-PP2-34) approved by the Vilnius Regional Biomedical Research Ethics Committee.

Both BMMSCs and chondrocytes were isolated and cultured according to our previous study [25]. Briefly, BMMSCs were isolated from the healthy human bone marrow samples (*n* = 5), received from Santaros Klinikos after joint surgery in Vilnius. Bone samples were washed with phosphate-buffered saline (PBS) (Sigma Aldrich), excluded from the bone, and chopped to liquid consistency in low glucose (1 g/L) Dulbecco’s modified Eagle’s medium (DMEM) (Capricorn Scientific, Germany). The obtained suspension was filtered through a 100 µm filter and centrifuged for 10 min at 350× *g*. The cell pellet was resuspended in DMEM medium, supplemented with 10% FBS (Gibco, Life Technologies, Grand Island, NY, USA) and 1% penicillin/streptomycin (PS) (Gibco, Life Technologies) (later referred to as complete DMEM medium), and fibroblast growth factor 2 (FGF-2) (20 ng/mL) (Thermo Fischer Scientific), counted and cultured in flasks under regular cell growth conditions.

Chondrocytes were isolated from post-operative human articular cartilage samples (*n* = 5), received from Santaros Klinikos after joint surgery. Cartilage samples were washed with PBS containing 1% PS solution and chopped into small pieces, with an average size of 1 mm^2^. Minced cartilage tissue was incubated in 1 g/L DMEM with 1% PS at 37 °C, and 5% CO_2_ overnight. After the incubation, cells from cartilage tissue samples were isolated enzymatically in pronase (Sigma Aldrich) solution for one hour at 37 °C and 5% CO_2_, later with type II collagenase solution (545 U/mL) (Biochrom AG) 10 milliliters/1 g of cartilage sample. Chondrocyte isolation was performed for 4 h at 37 °C and 5% CO_2_ under constant shaking. After, the cell suspension was centrifuged at 400× *g* for 5 min and the cell pellet was suspended in a complete DMEM medium. Isolated chondrocytes were cultured in flasks with a complete DMEM medium in a 37 °C incubator with 5% CO_2_. BMMSCs and chondrocyte complete medium was changed twice a week. For further experiments, the cells were detached using trypsin/EDTA 0.25% solution (Thermo Fischer Scientific) and counted using a hemocytometer.

### 2.3. Cell Proliferation Analysis

For proliferation analysis, BMMSCs and chondrocytes were seeded into 12 well plates, at a density of 20,000 cells/well, with/without Ppy and Ppy/Au NPs (10 μg/mL). Cell proliferation analysis was determined at 1, 3, and 7 days by commercially available Alamar blue (Thermo Fischer Scientific) according to the manufacturer’s recommendations. The Alamar blue kit allows measuring cell proliferation and/or the metabolic activity of the cells by producing the dye, which is reduced by intracellular dehydrogenases and produces a pink substrate easily measured by spectrophotometer (SpectraMax i3, Molecular Devices, San Jose, CA, USA) at 560/590 nm. The intensity of dye absorption generated by cells is directly proportional to the number of proliferating cells. Three technical replicates of three donor cells of each type were measured.

### 2.4. Cell Viability Test

BMMSC and chondrocyte viability were evaluated using a commercial Live/Dead cell viability assay (Thermo Fischer Scientific). The cells were seeded into 6 well plates, at a density of 20,000 cells/well, with/without Ppy and Ppy/Au NPs (10 μg/mL), and cultivated for 21 days. After the cells were stained with Live/Dead reagents, containing fluorescent Calcein-AM dye, which reacts with an intracellular esterase for alive cells, and ethidium homodimer-1, which intercalates into DNA of dead cells. The cells were then visualized with a fluorescent microscope (EVOS).

### 2.5. Chondrogenic Differentiation of Cells

Chondrogenic differentiation of cells was induced using a protocol routinely used at State Research Institute Centre for Innovative Medicine, Lithuania [26]. The chondrogenic medium consisted of high glucose (4.5 g/L) DMEM medium, 1% PS, 1% insulin-transferrin-selenium (Gibco Life Technologies), 350 μM L-proline (Carl Roth), 0.1% dexamethasone, 170 μM ascorbic acid-phosphate (Sigma Aldrich). For stimulating chondrogenic response, 10 ng/mL of TGF-β3 (Gibco, Life Technologies) was used.

The cells were differentiated in 2D and 3D models. For 2D differentiation, the cells were detached and 250,000 cells were seeded into 12 well plates with complete DMEM medium. The next day, after the cells are attached, the complete medium was changed to a chondrogenic medium containing or not NPs, and TGF-β3. For 3D differentiation, 250,000 cells were transferred into 15 mL tubes, centrifuged for 5 min, 500× *g*, and 250 μL of the chondrogenic medium was carefully applied on top of the cell pellet.

During chondrogenic differentiation, both, 2D and 3D cells were divided into six groups: 1. Without NPs (w/o NP); 2. Ppy NPs (10 μg/mL); 3. Ppy/Au NPs (10 μg/mL); 4. TGF-β3 (10 ng/mL); 5. TGF-β3 + Ppy NPs; 6. TGF-β3 + Ppy/Au NPs. The cells were differentiated for 21 days, changing the medium every other day.

### 2.6. COMP ELISA

Chondrogenic differentiation of BMMSCs and chondrocytes in 2D was evaluated by synthesized and released cartilage oligomeric matrix protein (COMP) after 21 days of chondrogenesis. Cell supernatants (3 days after the last medium change) were collected and the levels of COMP were estimated using COMP ELISA (Biovendor) according to the manufacturer’s instructions. The absorbance was measured at 450 nm using the spectrophotometer SpectraMax i3 (Molecular Devices, USA).

### 2.7. RNA Extraction from and RT-qPCR

After 2D chondrogenic differentiation, the cells were listed using LTR lysis buffer (Qiagen, 74104, Hilden, Germany) and RNA was extracted according to the manufacturer’s protocol. The RNA concentration and purity of all samples were measured with SpectraMax i3 (Molecular Devices, USA). RNA was reverse-transcribed with a Maxima cDNA synthesis kit including dsDNase treatment (Thermo Fischer Scientific). RT-qPCR reaction mixes were prepared with Maxima Probe qPCR Master Mix (Thermo Fischer Scientific) and TaqMan Gene expression Assays (RPS9–Hs02339424_g1, B2M–Hs00984230_m1, *COL2A1*–Hs01060345_m1, *ACAN*–Hs00153936_m1, *SOX9*–Hs00165814_m1 (Thermo Fischer Scientific), and ran on the Agilent Aria MX instrument (Agilent Technologies) in technical triplicates starting with denaturation step at 95 °C for 10 min followed by 40 cycles at 95 °C for 15 s of denaturation and 60 s for annealing and extension. Relative levels of gene transcripts were calculated by subtracting the threshold cycle (Ct) of the normalizer (the geometric mean of the two housekeeping genes RPS9 and B2M) from the Ct of the gene of interest, giving the dCt values that were subsequently transformed to 2-dCt values and multiplied by 1000 to scale-up for better graphical representation.

### 2.8. Histology and Immunohistochemistry

Histochemical analysis was applied to 3D differentiated cell pellets. The samples were fixed in 10% of neutral formalin and embedded into paraffin; 4 µm sections were deparaffinized and stained with safranin-O (Sigma Aldrich) (pH 2.0) for 3 min. The safranin-O stains negatively charged glycosaminoglycans (GAGs) pink/red. Stained sections were evaluated and blindly scored independently by two histology experts.

### 2.9. Statistical Analysis

The results are presented by the mean ± standard deviation (SD) from three repeats of not less than three cell cultures. Data are significant at a *p*-value of ≤0.05 calculated by Excel and GraphPad PRISM8.4.0 (455) software.

## 3. Results

### 3.1. AuNPs, Ppy, and Ppy/Au NPs Synthesis

**Figure 1:**
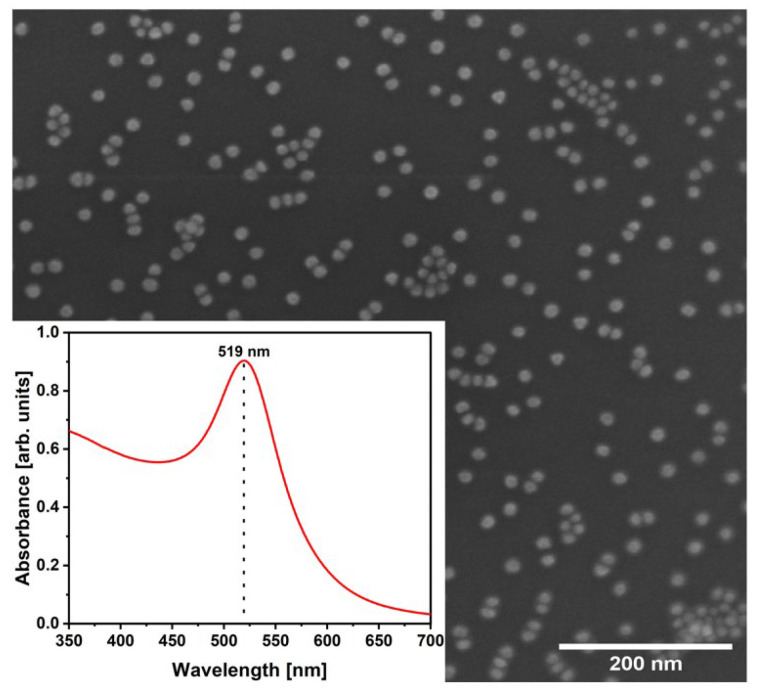
SEM image of synthesized AuNPs. Inset: UV-Vis spectra of AuNPs suspension.

The synthesized AuNPs were characterized before the preparation of Ppy/Au NPs. The AuNPs of regular round shape with size 13.66 ± 1.63 nm were observed using SEM (Figure 1). The λ_max_ of AuNPs was indicated at 519 nm. The prepared AuNPs were very similar to the NPs obtained previously [19].

**Figure 2:**
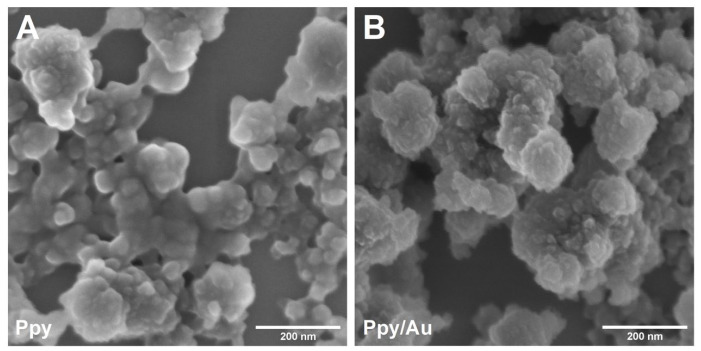
SEM images of (**A**) Ppy and (**B**) Ppy/Au NPs.

After 48 h of polymerization, black Ppy and Ppy/Au NPs solutions were monitored. Both solutions were washed by centrifugation to remove residual Ppy monomers and oligomers to obtain biocompatible NPs. The morphology of the NPs was studied using SEM (Figure 2). In both cases, nanostructured agglomerates/aggregates were monitored, where it is clearly seen that they are composed of smaller NPs. Individual AuNPs were not obtained on the surface of the Ppy/Au NPs sample, which can be described as evidence of their presence within the Ppy/Au NPs. AuNPs could possibly act as seeds for Ppy/Au NPs. The actual size of Ppy and Ppy/Au NPs is difficult to estimate. However, it is obvious that Ppy/Au NPs are smaller than Ppy NPs.

### 3.2. Cell Morphology, Proliferation, and Viability after Incubation with Ppy and Ppy/Au NPs

**Figure 3:**
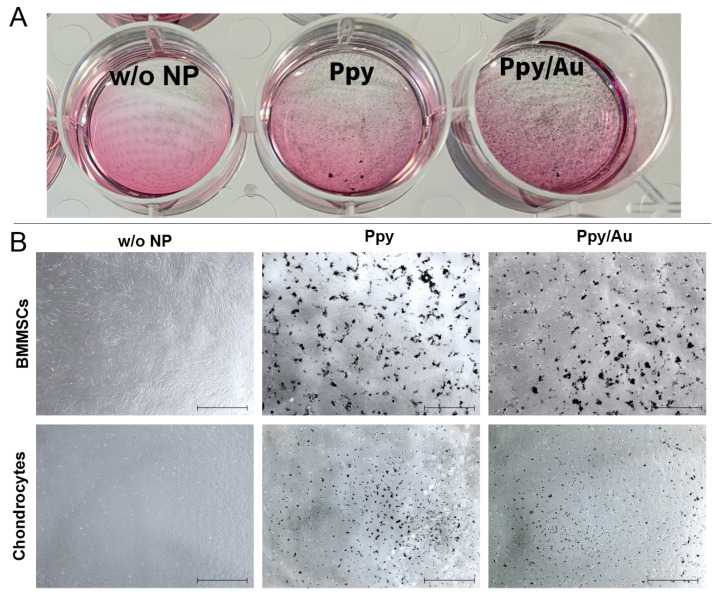
BMMSCs and chondrocytes cultivated without and with Ppy or Ppy/Au NPs (10 μg/mL) for 7 days. (**A**): macroscopic view of the cell plate. (**B**): cells visualized under light microscopy, X40. w/o NP control cells, cultivated under the same conditions, but without NPs.

BMMSCs were characterized according to stem cell properties: surface markers and ability to differentiate into adipogenic, osteogenic lineages, as published before [25,26]. BMMSCs and chondrocytes were cultivated for 7 days with Ppy and Ppy/Au NPs and visualized under a light microscope for evaluating cell morphology and Ppy NPs distribution (Figure 3).

**Figure 4:**
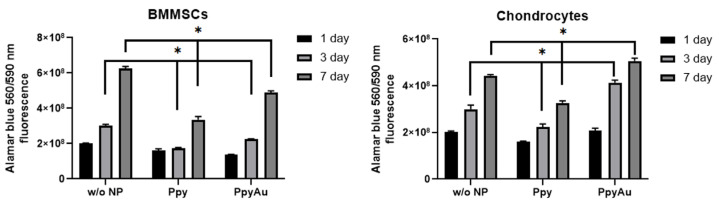
The proliferation of BMMSCs and chondrocytes cultivated with and without Ppy and Ppy/Au NPs (10 μg/mL) after 1, 3 and 7 days, measured using Alamar blue dye, which fluorescence measured with a spectrophotometer at 560/590 nm. w/o NP control cells, cultivated under the same conditions, but without NPs (*n* = 3). Data are presented as mean ± SD. * Horizontal bars represent *p* ≤ 0.05.

It was observed that Ppy and Ppy/Au NPs cover cell monolayers and remain stable during the whole cultivation period. Cell proliferation was analyzed after 1, 3 and 7 days by measuring Alamar blue dye fluorescence, to evaluate cell metabolic activity and proliferation sensitivity to Ppy NPs (Figure 4).

It was noticed that BMMSCs are more sensitive to Ppy NPs than chondrocytes. Ppy and Ppy/Au NPs significantly inhibited BMMSCs proliferation after 3 and 7 days. Chondrocytes demonstrated different effects of Ppy and Ppy/Au NPs, where incubation with Ppy NPs resulted in significantly lower proliferation after 3 and 7 days, while Ppy/Au NPs significantly increased chondrocyte growth after 3 and 7 days.

**Figure 5:**
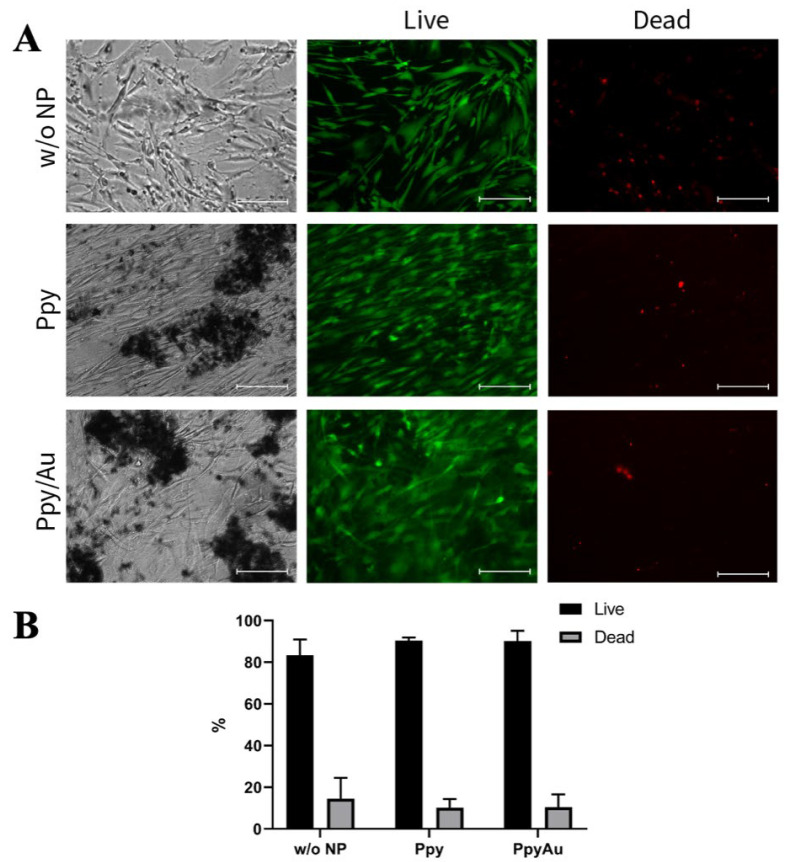
BMMSC viability after cultivating with/without Ppy and Ppy/Au NPs (10 μg/mL) for 21 days, after stained with viability dyes calcein-green for live cells and propidium iodide for dead cells (Live/Dead kit). (**A**): cells visualized under fluorescent microscopy, using blue (488 nm) and green (532 nm) lasers, ×100. (**B**): quantified percentage of Live/Dead cells. w/o NP control cells, cultivated under the same conditions, but without NPs.

For the cell viability test, BMMSCs were incubated with Ppy and Ppy/Au NPs for 21 days, a period required for chondrogenic differentiation, and stained with a Live/Dead kit (Figure 5). According to viability dyes, most of the BMMSCs remained viable after 21 days with Ppy and Ppy/Au NPs, as compared to control cells without NPs.

### 3.3. Chondrogenic Differentiation after Incubation with Ppy and Ppy/Au NPs

#### 3.3.1. Cartilage Oligomeric Matrix Protein Level in Cell Supernatants was Higher after Incubation with Ppy and Ppy/Au NPs

**Figure 6:**
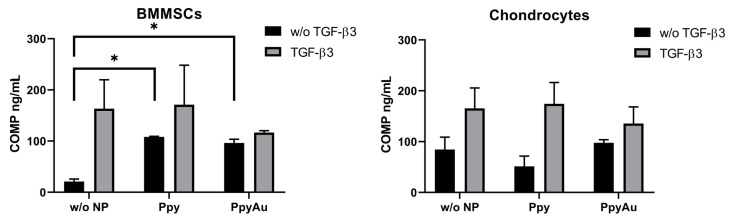
Levels of COMP in BMMSCs and chondrocytes medium after 21 days of chondrogenic differentiation in monolayer (ELISA), with TGF-β3 (10 ng/mL), Ppy (10 μg/mL), Ppy/Au NPs (10 μg/mL), as well as without TGF-β3 (w/o TGF-β3) and without NPs (w/o NP). Data are presented as mean ± SD. * Horizontal bars represent *p* ≤ 0.05.

COMP was measured in cell supernatants after chondrogenic induction. COMP may be used as an indicator for chondrogenic differentiation in cells and its synthesis reveals the formation of the cartilage extracellular matrix (ECM) [27]. Levels of secreted COMP by BMMSCs and chondrocytes after chondrogenic induction were measured in cell supernatants (Figure 6).

There was no significant change in chondrocyte COMP secretion; however, a significant increase in COMP levels was observed in BMMSCs samples stimulated with Ppy and Ppy/Au NPs in the absence of TGF-β3. No significant differences were observed between the effects of Ppy and Ppy/Au groups. This result indicates the stimulatory effects of Ppy and Ppy/Au NPs on BMMSCs even without important growth factors for chondrogenesis, i.e., TGF-β3.

#### 3.3.2. Chondrogenic Gene Expression in Cells was Higher after Incubation with Ppy and Ppy/Au NPs

**Figure 7:**
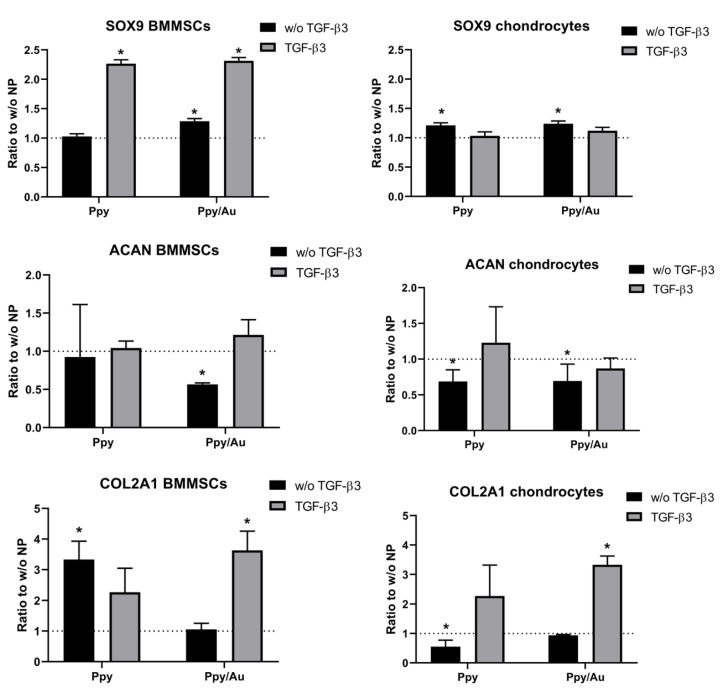
Chondrogenic gene (*SOX9*, *ACAN*, *COL2A1*) expression in BMMSCs and chondrocytes, after 21 days of chondrogenic differentiation in monolayer with TGF-β3 (10 ng/mL), Ppy (10 μg/mL), Ppy/Au NPs (10 μg/mL), as well as without TGF-β3 (w/o TGF-β3) and without NPs (w/o NP). Relative mRNA level was normalized to two housekeeping genes (B2M and RPS9) and expressed as 2−ΔCt*1000, the ratio of Ppy and Ppy/Au NP stimulated samples vs. non-stimulated controls. Data are presented as mean ± SD. * represent differences *p* ≤ 0.05, which was calculated vs. controls without NPs.

After 21 days of chondrogenic induction, cartilage-specific gene expression was analyzed in chondrocytes and BMMSCs. Aggrecan (*ACAN*), collagen type II (*COL2A1*), and early chondrogenesis marker transcription factor *SOX9* are presented in Figure 7.

*SOX9* gene expression was upregulated by Ppy and Ppy/Au NPs in both cell types, as compared to non-stimulated with NPs controls. Chondrocytes *SOX9* gene expression was significantly higher in both, Ppy and Ppy/Au NPs samples without TGF-β3 stimulation, whereas in BMMSCs application of Ppy and Ppy/Au NPs resulted in a significant increase of *SOX9* in the TGF-β3 stimulated group. Additionally, Ppy/Au NPs significantly upregulated *SOX9* in BMMSCs even in the absence of TGF-β3, similarly as in chondrocytes.

Aggrecan gene (*ACAN*) expression was downregulated in BMMSCs samples stimulated by Ppy and Ppy/Au NPs without TGF-β3; in Ppy/Au NPs samples this difference was significant, as compared to samples without NPs. In chondrocytes, the *ACAN* gene was downregulated after incubation with Ppy and Ppy/Au NPs. No significant differences were observed in samples with TGF-β3.

Collagen type II is the most robust type of collagens present in cartilage and according to its gene expression, Ppy and Ppy/Au NPs significantly upregulated its expression in BMMSCs, as compared to control samples incubated without NPs. Important to note, that the *COL2A1* gene was upregulated more in samples without TGF-β3, which is similar to COMP secretion in BMMSCs (Figure 6). In chondrocytes, Ppy in the absence of TGF-β3 downregulated *COL2A1* gene expression, while Ppy/Au NPs to TGF-β3 stimulated samples significantly increased *COL2A1* expression, as compared to non-stimulated with NPs controls.

Thus, even though both Ppy and Ppy/Au NPs stimulated BMMSCs chondrogenesis, these cells were more responsive to Ppy, than to Ppy/Au NPs, as Ppy effects were more pronounced even in the absence of TGF-β3. Chondrocytes expressed more *COL2A1* in response to Ppy/Au NPs.

#### 3.3.3. Chondrogenic Differentiation in Pellets after Incubation with Ppy and Ppy/Au NPs

**Figure 8:**
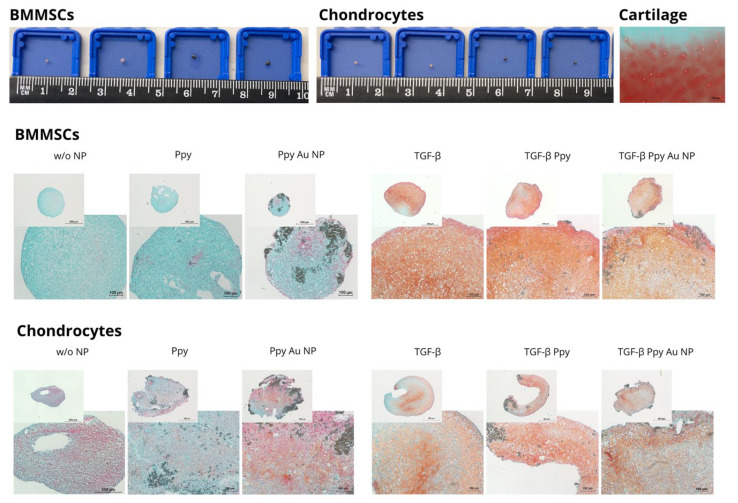
Chondrogenic differentiation of BMMSCs and chondrocytes (macroscopic and microscopic views). Cells were incubated in a chondrogenic differentiation medium with/without TGF-β3 (10 ng/mL) and with/without Ppy (10 μg/mL), Ppy/Au NPs (10 μg/mL) for 21 days, after cell pellets were histologically analysed. Histological sections of cell pellets, stained with safranin-O and visualized under light microscopy ×40/×100 magnification. w/o NP control cells without NPs. Cartilage positive control staining for cartilage histological sections with safranin-O.

Histological samples of BMMSC and chondrocyte pellets after 21 days of chondrogenic differentiation, stained with safranin-O (for cartilage ECM proteins) are presented in Figure 8, with the most representative samples. In accordance with gene expression, incubation with Ppy and Ppy/Au stimulated the production of ECM in BMMCSs and chondrocytes even in the absence of TGF-β3, which is visible by Safranin O dye staining by pinkish-red colored GAGs. Adding TGF-β3 resulted in a much stronger production of GAGs in BMMSCs and chondrocytes. It is important to note that the BMMSCs stimulated with TGF-β3 and Ppy possessed the strongest ECM (GAG) formation, as compared to other samples. Similar effects were observed in BMMSCs chondrogenic gene expression profile, where single Ppy stimulation resulted in an upregulated of the *COL2A1* gene similar to TGF-β3 stimulation. Chondrocytes showed stronger ECM synthesis in samples stimulated with Ppy/Au NPs without TGF-β3 (Figure 8), while Ppy tended to decrease ECM without TGF-β3, as compared to samples without NPs. These results correspond to gene expression results.

## 4. Discussion

Conductive nanomaterials have attracted a lot of researchers’ attention and have been used in different applications, including biomedicine and tissue regeneration. Ppy is one of the most studied polymers due to its biocompatibility, easy synthesis and ease of application in different approaches [28,29]. Ppy can be combined with different molecules, including organic compounds and metal NPs, as well as incorporated into synthetic or organic scaffolds/hydrogels, used as layers/films/membranes. Ppy constructs are usually tested in combination with ES, as an additional charge conductor. Ppy/ES platform has been studied in different in vivo and in vitro systems, stimulating cell/tissue functions or introducing the drugs. However, most of these studies were focused on muscle, nerve and cardiac tissue functions, but not cartilage [30,31,32].

Cartilage is a dense, avascular tissue with a very low number of cells, which makes it highly susceptible to vast diseases such as OA. In previous studies, ES was used to stimulate chondrocyte functions and even boost BMMSCs ability to differentiate into the chondrogenic lineage, as these cells are the most potential candidates to be used for cartilage regeneration. However, according to our knowledge, chondrogenic differentiation of BMMSCs using Ppy NPs has never been studied before, except for the development of Ppy-hydrogels for cartilage tissue engineering [32].

In this study, we analyzed BMMSCs and chondrocyte functions after incubating with Ppy and Ppy/Au NPs and evaluated the chondrogenic differentiation capacity of both cell types. First of all, we incubated the cells in cell culture plates with Ppy and Ppy/Au NPs and observed cell morphology, as well as their proliferation and viability. As expected, Ppy and Ppy/Au NPs covered the cell monolayer; however, the cells remained viable even after 21 days of incubation with NPs. Many studies tested different Ppy-based platforms on cell viability using a Live/Dead kit and reported that cell viability was not attenuated. For instance, Ppy-nanorods used for MC3T3-E1 (mouse calvaria-derived osteoblast precursor cells), or PC12 cells (pheochromocytoma of rat adrenal medulla-derived cell line) cell lines did not affect cell viability and were not cytotoxic [33]. Similar viability was observed in endothelial and cardiac progenitor cells after seeding them to Ppy films [34] and neural stem cells seeded on collagen/Ppy scaffolds [35], indicating good biocompatibility of Ppy structures.

Despite a positive viability test, BMMSC and chondrocyte proliferation capacity with Ppy and Ppy/Au NPs demonstrated differences. Both, Ppy and Ppy/Au NPs decreased the proliferation of BMMSCs, while only Ppy NPs decreased chondrocyte proliferation after 3 and 7 days. Most of the studies made before support MSC proliferation on Ppy-films/layers/hydrogels [36,37,38]; however, in our case, we used Ppy and Ppy/Au NPs, which were demonstrated to not have any cytotoxic effects on mice peritoneum cells in vivo [38]. The decreased proliferation rate of cells might be caused due to particles covering the cell monolayer, which affects their metabolic activity, but not viability.

Chondrogenesis of BMMSCs and chondrocytes was stimulated in two different models, monolayer (2D) and classical pellets (3D). To verify chondrogenic differentiation in cells, synthesis, and secretion of COMP were measured in cell supernatants after chondrogenic differentiation. COMP or thrombospondin-5, is a classical cartilage ECM biomarker [27]. We observed a significant increase of COMP in BMMSCs Ppy and Ppy/Au NPs samples without TGF-β3. This indicates that Ppy NPs stimulate the cell response for the production of COMP, even without classical chondrogenesis stimulating growth factor TGF-β3, which activates chondrogenesis in cells [39]. The mechanism of Ppy and Ppy/Au NPs actions is still under investigation; however, several hypotheses link their effect to electrophysiological modulation, and hyperpolarizing of the cell membrane, which leads to activation of voltage-gated Ca^2+^ channels and voltage-gated sodium channels, as described before [7]. MSCs, including BMMSCs are often used in various systems to stimulate their cellular changes through ion channels in order to bioelectrically support specific tissue formation. Most of the non-excitable cells express a wide range of ion channels, including voltage-gated Ca^2+^ channels and voltage-gated sodium channels. Ca^2+^ ions are known to be important for stimulating chondrogenic response, such as ECM production, and activation of Ca^2+^ dependant transcription factors (*SOX9*) [40,41].

Chondrogenic gene expression was also upregulated in BMMSCs and chondrocytes after incubation with Ppy and Ppy/Au NPs, as compared to the non-stimulated control. Transcription factor *SOX9* is important for further induction of cartilage ECM matrix protein synthesis. Ppy and Ppy/Au NPs significantly upregulated *SOX9* gene expression in BMMSCs after incubation with TGF-β3, while in chondrocytes *SOX9* was upregulated by Ppy and Ppy/Au NPs in the absence of TGF-β3, as compared to non-stimulated with NPs samples. *ACAN* gene expression was upregulated in BMMSCs Ppy/Au NPs together with TGF-β3, whereas in chondrocytes it was upregulated in Ppy with TGF-β3 samples. Collagen type II (*COL2A1* gene), which is the most abundant type of collagen in cartilage was also upregulated in both cell types. In chondrocytes, collagen type II expression was significantly higher after stimulation with TGF-β3 and Ppy/Au NPs samples, while in BMMSCs, Ppy resulted in strong upregulation of *COL2A1* without TGF-β3, as compared to non-stimulated with Ppy NPs control. Therefore, BMMSCs were more sensitive and responsive to Ppy NPs, than Ppy/Au NPs, while chondrocytes were more responsive to Ppy/Au NPs. Histological pellet analysis also revealed similar results as gene expression. Production of cartilage proteoglycans was observed after staining histological sections safranin-O in Ppy and Ppy/Au NPs samples, even without TGF-β3 stimulation in BMMSCs. In chondrocytes, proteoglycans were more abundantly produced in samples stimulated with Ppy/Au NPs. As mentioned before, Ppy or Ppy/Au NPs were never used before for chondrogenic differentiation studies of MSCs; however, Ppy-based structures were used for stimulating cardiac, neural, osteogenic, and muscle differentiation in MSCs and showed promising results. For instance, Ppy films increased the osteogenic differentiation of rat BMMSCSs by enhancing calcium deposition and ECM mineralization [42]. Additionally, Poly(lactic-co-glycolic acid) fiber scaffold coated with Ppy was used for iPSC cardiogenic differentiation and it increased expression of cardiac genes (Actinin, NKX2.5, GATA4, Myh6) and decreased the level of stemness factors (Oct4, Nanog) [31]. Ppy/chondroitin sulfate, Ppy/dextran sulfate and Ppy/(dodecyl benzene sulfonic acid increased the proliferation and differentiation of primary mice skeletal muscle myoblasts [43] and the Ppy coated poly(trimethylene carbonate) scaffold increased proliferation and enhanced expression of calponin, myosin heavy chain and smooth muscle actin in human adipose stem cells [44].

Thus, the diverse effects of Ppy and Ppy/Au NPs on BMMSCs and chondrocytes bring novel insights for in vitro differentiation and cartilage tissue ECM synthesis using conductive polymers. In addition to that, Ppy has been shown to be efficient in drug delivery or sustained active release of molecules, such as growth factors, which are crucial for the chondrogenesis of stem cells. For instance, Ppy-coated polyvinylidene fluoride fibers were used as an electrosensitive growth factor release system for nerve growth factor [45]. Ppy combined with bone morphogenetic proteins has shown the potential to promote osteogenic differentiation of MC3TC-E1 cells in light-to-heat photothermal systems [46]. Polydopamine-Ppy microcapsules or Ppy-coated nanostructured electrodes can be used as an on-demand release system of the anti-inflammatory drug dexamethasone as a potential approach for rheumatic diseases [47,48]. The controlled release of the non-steroidal anti-inflammatory drug ibuprofen from Ppy films was also demonstrated [49]. Therefore, the broad application of Ppy and its combination with drugs/growth factors is a promising area for further chondrogenesis-related studies and cartilage tissue repair.

## 5. Conclusions

In conclusion, this study presents novel data on the potential effects of Ppy and Ppy/Au NPs for BMMSCs chondrogenic differentiation in vitro, even without applying ES, which is important information for further cartilage regeneration protocols using ES-based techniques. We demonstrated the effects of Ppy NPs in stimulating chondrogenic response in human BMMSCs, while chondrocytes possessed stronger chondrogenic gene expression response in the presence of Ppy/Au NPs.

## Data Availability

The data supporting these findings can be found at State Research Institute Centre for Innovative Medicine, Department of Regenerative Medicine.

## Abbreviations

ACAN	aggrecan gene
Au NPs	gold NPs
BMMSCs	bone marrow mesenchymal stem cells
CCK-8	cell counting kit 8
COL2A1	collagen type II gene
COMP	cartilage oligomeric matrix protein
DMEM	Dulbecco’s modified Eagle’s medium
ECM	extracellular matrix
ES	electrical stimulation
FBS	fetal bovine serum
FGF2	fibroblast growth factor-2
MSCs	mesenchymal stem cells
NCDCs	nasal crest-derived chondrocytes
NP	nanoparticle
OA	osteoarthritis
PBS	phosphate-buffered saline
PFA	paraformaldehyde
SD	standard deviation
TGF-β3	transforming growth factor β3
Ppy	polypyrrole
Ppy NPs	polypyrrole nanoparticles
Ppy/Au NPs	polypyrrole/gold nanocomposites

## References

[1] German N., Ramanaviciene A., Ramanavicius A. (2019). Formation of polyaniline and polypyrrole nanocomposites with embedded glucose oxidase and gold nanoparticles. Polymers.

[2] Kaur G., Adhikari R., Cass P., Bown M., Gunatillake P. (2015). Electrically conductive polymers and composites for biomedical applications. RSC Adv..

[3] Fadel M., Fadeel D.A., Ibrahim M., Hathout R.M., El-Kholy A.I. (2020). One-step synthesis of polypyrrole-coated gold nanoparticles for use as a photothermally active nano-system. Int. J. Nanomed..

[4] Ateh D.D., Navsaria H.A., Vadgama P. (2006). Polypyrrole-based conducting polymers and interactions with biological tissues. J. R. Soc. Interface.

[5] Borges M.H.R., Nagay B.E., Costa R.C., Souza J.G.S., Mathew M.T., Barão V.A.R. (2023). Recent advances of polypyrrole conducting polymer film for biomedical application: Toward a viable platform for cell-microbial interactions. Adv. Colloid Interface Sci..

[6] Liu L., Li P., Zhou G., Wang M., Jia X., Liu M., Niu X., Song W., Liu H., Fan Y. (2013). Increased proliferation and differentiation of pre-osteoblasts MC3T3-E1 cells on nanostructured polypyrrole membrane under combined electrical and mechanical stimulation. J. Biomed. Nanotechnol..

[7] Liang Y., Goh J.C. (2020). Polypyrrole-incorporated conducting constructs for tissue engineering applications: A Review. Bioelectricity.

[8] Pelto J., Björninen M., Pälli A., Talvitie E., Hyttinen J., Mannerström B., Suuronen Seppanen R., Kellomäki M., Miettinen S., Haimi S. (2013). Novel polypyrrole-coated polylactide scaffolds enhance adipose stem cell proliferation and early osteogenic differentiation. Tissue Eng. Part A.

[9] Zhang J., Li M., Kang E.T., Neoh K.G. (2016). Electrical stimulation of adipose-derived mesenchymal stem cells in conductive scaffolds and the roles of voltage-gated ion channels. Acta Biomater..

[10] Björninen M., Siljander A., Pelto J., Hyttinen J., Kellomäki M., Miettinen S., Seppänen R., Haimi S. (2014). Comparison of chondroitin sulfate and hyaluronic acid doped conductive polypyrrole films for adipose stem cells. Ann. Biomed. Eng..

[11] Harrell C.R., Markovic B.S., Fellabaum C., Arsenijevic A., Volarevic V. (2019). Mesenchymal stem cell-based therapy of osteoarthritis: Current knowledge and future perspectives. Biomed. Pharmacother..

[12] Kalamegam G., Memic A., Budd E., Abbas M., Mobasheri A. (2018). A comprehensive review of stem cells for cartilage regeneration in osteoarthritis. Adv. Exp. Med. Biol..

[13] Jang S., Lee K., Ju J.H. (2021). Recent updates of diagnosis, pathophysiology, and treatment on osteoarthritis of the knee. Int. J. Mol. Sci..

[14] Johnstone B., Hering T.M., Caplan A.I., Goldberg V.M., Yoo J.U. (1998). In vitro chondrogenesis of bone marrow-derived mesenchymal progenitor cells. Exp. Cell Res..

[15] Farooqi A.R., Bader R., Van Rienen U. (2019). Numerical study on electromechanics in cartilage tissue with respect to its electrical properties. Tissue Eng. Part B Rev..

[16] Vaiciuleviciute R., Uzieliene I., Bernotas P., Novickij V., Alaburda A., Bernotiene E. (2023). Electrical stimulation in cartilage tissue engineering. Bioengineering.

[17] Mobasheri A., Matta C., Uzielienè I., Budd E., Martín-Vasallo P., Bernotiene E. (2019). The chondrocyte channelome: A narrative review. Jt. Bone Spine.

[18] Parate D., Franco-Obregón A., Fröhlich J., Beyer C., Abbas A.A., Kamarul T., Hui J.H.P., Yang Z. (2017). Enhancement of mesenchymal stem cell chondrogenesis with short-term low intensity pulsed electromagnetic fields. Sci. Rep..

[19] Mikoliunaite L., Kubiliute R., Popov A., Voronovič J., Šakirzanovas S., Ramanavičiene A., Ramanavičius A. (2014). Development of gold nanoparticle-polypyrrole nanocomposites. Chemija.

[20] Kumar P.P.P., Lim D.K. (2022). Gold-polymer nanocomposites for future therapeutic and tissue engineering applications. Pharmaceutics.

[21] Aoki T., Tanino M., Sanui K., Ogata N., Kumakura K. (1996). Secretory function of adrenal chromaffin cells cultured on polypyrrole films. Biomaterials.

[22] Wang X., Gu X., Yuan C., Chen S., Zhang P., Zhang T., Yao J., Chen F., Chen G. (2004). Evaluation of biocompatibility of polypyrrole in vitro and in vivo. J. Biomed. Mater. Res. Part A.

[23] Ramanaviciene A., Voronovic J., Popov A., Drevinskas R., Kausaite-Minkstimiene A., Ramanavicius A. (2016). Investigation of biocatalytic enlargement of gold nanoparticles using dynamic light scattering and atomic force microscopy. Colloids Surfaces A Physicochem. Eng. Asp..

[24] Leonavicius K., Ramanaviciene A., Ramanavicius A. (2011). Polymerization model for hydrogen peroxide initiated synthesis of polypyrrole nanoparticles. Langmuir.

[25] Uzieliene I., Bironaite D., Pachaleva J., Bagdonas E., Sobolev A., Tsai W.B., Kvedaras G., Bernotiene E. (2023). Chondroitin sulfate-tyramine-based hydrogels for cartilage tissue repair. Int. J. Mol. Sci..

[26] Uzieliene I., Bagdonas E., Hoshi K., Sakamoto T., Hikita A., Tachtamisevaite Z., Rakauskiene G., Kvederas G., Mobasheri A., Bernotiene E. (2021). Different phenotypes and chondrogenic responses of human menstrual blood and bone marrow mesenchymal stem cells to activin A and TGF-β3. Stem Cell Res. Ther..

[27] Haleem-smith H., Calderon R., Song Y., Tuan R.S., Faye H. (2013). Cartilage oligomeric matrix protein enhances. J. Cell. Biochem..

[28] Kumar A.M., Suresh B., Das S., Obot I.B., Adesina A.Y., Ramakrishna S. (2017). Promising bio-composites of polypyrrole and chitosan: Surface protective and in vitro biocompatibility performance on 316L SS implants. Carbohydr. Polym..

[29] Zare E.N., Agarwal T., Zarepour A., Pinelli F., Zarrabi A., Rossi F., Ashrafizadeh M., Maleki A., Shahbazi M.-A., Maiti T.K. (2021). Electroconductive multi-functional polypyrrole composites for biomedical applications. Appl. Mater. Today.

[30] Golabi M., Turner A.P.F., Jager E.W.H. (2016). Tuning the surface properties of polypyrrole films for modulating bacterial adhesion. Macromol. Chem. Phys..

[31] Gelmi A., Cieslar-Pobuda A., de Muinck E., Los M., Rafat M., Jager E.W.H. (2016). Direct mechanical stimulation of stem cells: A beating electromechanically active scaffold for cardiac tissue engineering. Adv. Healthc. Mater..

[32] Distler T., Polley C., Shi F., Schneidereit D., Ashton M.D., Friedrich O., Kolb J.F., Hardy J.G., Detsch R., Seitz H. (2021). Electrically conductive and 3D-printable oxidized alginate-gelatin polypyrrole: PSS Hydrogels for Tissue Engineering. Adv. Healthc. Mater..

[33] Bhattarai D.P., Hwang T.I., Kim J.I., Lee J.H., Chun S., Kim B.S., Park C.H., Kim C.S. (2020). Synthesis of polypyrrole nanorods via sacrificial removal of aluminum oxide nanopore template: A study on cell viability, electrical stimulation and neuronal differentiation of PC12 cells. Mater. Sci. Eng. C.

[34] Gelmi A., Ljunggren M.K., Rafat M., Jager E.W.H. (2014). Influence of conductive polymer doping on the viability of cardiac progenitor cells. J. Mater. Chem. B.

[35] Xu X., Wang L., Jing J., Zhan J., Xu C., Xie W., Ye S., Zhao Y., Zhang C., Huang F. (2022). Conductive collagen-based hydrogel combined with electrical stimulation to promote neural stem cell proliferation and differentiation. Front. Bioeng. Biotechnol..

[36] Castano H., O’Rear E.A., McFetridge P.S., Sikavitsas V.I. (2004). Polypyrrole thin films formed by admicellar polymerization support the osteogenic differentiation of mesenchymal stem cells. Macromol. Biosci..

[37] Maharjan B., Kaliannagounder V.K., Jang S.R., Awasthi G.P., Bhattarai D.P., Choukrani G., Park C.H., Kim C.S. (2020). In-situ polymerized polypyrrole nanoparticles immobilized poly(ε-caprolactone) electrospun conductive scaffolds for bone tissue engineering. Mater. Sci. Eng. C.

[38] Ramanaviciene A., Kausaite A., Tautkus S., Ramanavicius A. (2010). Biocompatibility of polypyrrole particles: An in-vivo study in mice. J. Pharm. Pharmacol..

[39] Kim M., Ericksona I.E., Choudhurya M., Pleshkoc N., Maucka R.L. (2012). Transient exposure to TGF-β3 improves the functional chondrogenesis of MSC-laden hyaluronic acid hydrogels Minwook. J. Mech. Behav. Biomed. Mater..

[40] Maumus M., Fonteneau G., Ruiz M., Assou S., Boukhaddaoui H., Pastoureau P., De Ceuninck F., Jorgensen C., Noel D. (2021). Neuromedin B promotes chondrocyte differentiation of mesenchymal stromal cells via calcineurin and calcium signaling. Cell Biosci..

[41] Matta C., Zakany R. (2013). Calcium signalling in chondrogenesis: Implications for cartilage repair. Front. Bioeng. Biotechnol..

[42] Li C., Hsu Y.T., Hu W.W. (2016). The regulation of osteogenesis using electroactive polypyrrole films. Polymers.

[43] Gilmore K.J., Kita M., Han Y., Gelmi A., Higgins M.J., Moulton S.E., Clark G.M., Kapsa R., Wallace G.G. (2009). Skeletal muscle cell proliferation and differentiation on polypyrrole substrates doped with extracellular matrix components. Biomaterials.

[44] Björninen M., Gilmore K., Pelto J., Seppänen-Kaijansinkko R., Kellomäki M., Miettinen S., Wallace G., Grijpma D., Haimi S. (2017). Electrically stimulated adipose stem cells on polypyrrole-coated scaffolds for smooth muscle tissue engineering. Ann. Biomed. Eng..

[45] Miar S., Ong J.L., Bizios R., Guda T. (2021). Electrically stimulated tunable drug delivery from polypyrrole-coated polyvinylidene fluoride. Front. Chem..

[46] Ulasevich S., Ryzhkov N.V., Andreeva D.V., Özden D.S., Piskin E., Skorb E.V. (2020). Light-to-Heat photothermal dynamic properties of polypyrrole-based coating for regenerative therapy and lab-on-a-chip applications. Adv. Mater. Interfaces.

[47] Xie C., Li P., Han L., Wang Z., Zhou T., Deng W., Wang K., Lu X. (2017). Electroresponsive and cell-affinitive polydopamine/polypyrrole composite microcapsules with a dual-function of on-demand drug delivery and cell stimulation for electrical therapy. NPG Asia Mater..

[48] Leprince L., Dogimont A., Magnin D., Demoustier-Champagne S. (2010). Dexamethasone electrically controlled release from polypyrrole-coated nanostructured electrodes. J. Mater. Sci. Mater. Med..

[49] Justin G.A., Zhu S., Nicholson T.R., Maskrod J., Mbugua J., Chase M., Jung J.H., Mercado R.M.L. (2012). On-demand controlled release of anti-inflammatory and analgesic drugs from conducting polymer films to aid in wound healing. Conf. Proc. IEEE Eng. Med. Biol. Soc..

